# A novel method to monitor rheumatoid arthritis prevalence using hospital and medication databases

**DOI:** 10.1186/s13075-024-03366-x

**Published:** 2024-07-16

**Authors:** Louise Koller-Smith, Ahmed Mehdi, Lyn March, Leigh Tooth, Gita D. Mishra, Ranjeny Thomas

**Affiliations:** 1 Frazer Institute, The University of Queensland, Translational Research Institute (TRI), Level 7, 37 Kent St, Woolloongabba, QLD 4102 Australia; 2 Florance and Cope Professorial Department of Rheumatology, Royal North Shore Hospital and Kolling Institute and Sydney MSK Flagship, Faculty of Medicine and Health, University of Sydney, Sydney, NSW Australia; 3 Facility for Advanced Bioinformatics, Queensland Cyber Infrastructure Foundation Ltd, Brisbane, QLD 4072 Australia; 4 School of Public Health, The University of Queensland, Brisbane, QLD Australia

**Keywords:** Rheumatoid arthritis, Self-report, Prevalence, Case-finding, Database

## Abstract

**Background:**

Most estimates of rheumatoid arthritis (RA) prevalence, including all official figures in Australia and many other countries, are based on self-report. Self-report has been shown to overestimate RA, but the ‘gold standard’ of reviewing individual medical records is costly, time-consuming and impractical for large-scale research and population monitoring. This study provides an algorithm to estimate RA cases using administrative data that can be adjusted for use in multiple contexts to provide the first approximate RA cohort in Australia that does not rely on self-report.

**Methods:**

Survey data on self-reported RA and medications from 25 467 respondents of the Australian Longitudinal Study on Women’s Health (ALSWH) were linked with data from the national medication reimbursement database, hospital and emergency department (ED) episodes, and Medicare Benefits codes. RA prevalence was calculated for self-reported RA, self-reported RA medications, dispensed RA medications, and hospital/ED RA presentations. Linked data were used to exclude individuals with confounding autoimmune conditions.

**Results:**

Of 25 467 survey respondents, 1367 (5·4%) women self-reported disease. Of the 26 840 women with hospital or ED presentations, 292 (1·1%) received ICD-10 codes for RA. There were 1038 (2·8%) cases by the medication database definition, and 294 cases (1·5%) by the self-reported medication definition. After excluding individuals with other rheumatic conditions, prevalence was 3·9% for self-reported RA, 1·9% based on the medication database definition and 0·5% by self-reported medication definition. This confirms the overestimation of RA based on self-reporting.

**Conclusions:**

We provide an algorithm for identifying individuals with RA, which could be used for population studies and monitoring RA in Australia and, with adjustments, internationally. Its balance of accuracy and practicality will be useful for health service planning using relatively easily accessible input data.

**Supplementary Information:**

The online version contains supplementary material available at 10.1186/s13075-024-03366-x.

## Background

The inflammatory autoimmune disease rheumatoid arthritis (RA) affects 23 million people worldwide, over half of whom are of working age [1]. RA is a significant cause of disability, negatively affecting quality of life, ability to care for self and others, and workforce participation, and reduces lifespan by approximately 10 years [2]. Currently, RA is an incurable disease, and long-term treatments for RA carry potential serious toxicities including increased risk of fractures, diabetes and infections. Information on RA incidence and prevalence is required to allocate resources and effectively analyse the cost-benefit of emerging disease interventions, but the human and economic burden of RA in Australia and many other countries has not been accurately quantified. In fact, most estimates of RA prevalence, including all official figures in Australia, are based on self-reported cases obtained via survey [3]. Large population cohort studies are an important source of data predicting risk factors and symptoms of rheumatoid arthritis (RA), and such studies often use self-reported data on whether the patient has RA, in order to assign cohorts.

Self-reported RA is expected to have poor validity because patients may not know the subtype of arthritis they have (particularly osteoarthritis versus rheumatoid arthritis) or may classify any musculoskeletal pain as arthritis [4–6]. This expectation has been confirmed in international validation studies, which compare self-reported RA with a gold standard - usually medical record review or expert opinion. A large study from Norway found that only 19.1% of 2703 self-reported RA cases were true positives [7], while a study of women of high educational background showed that self-reported RA was confirmed by medical record review in only 35.8% of cases [8]. Poor validity has consistently been found to be due to over-reporting of RA (false positives) rather than under-reporting (false negatives) [7, 9], meaning that future studies should focus on validating self-reported cases with the addition of other information such as prescribed medications, rather than on searching for missed cases that are not apparent from self-report. In line with this, validation studies combining self-reported diagnosis with self-reported medication use, symptom-specific questionnaires or admissions data have improved positive predictive values (PPV) to up to 90% [10, 11]. A systematic review from 2013 of claims-based algorithms for RA case-finding found that algorithms performed better if they used at least two ICD/procedure codes, included medications, or required participation of a rheumatologist in patient care [12]. A more valid method of determining RA cases for use in population studies is needed, as ascertainment bias with many false positive cases will dilute the effect of differences between RA cases and healthy controls so that associations may not be detected in population studies using self-reported RA [7].

Large scale validation of RA is more difficult for RA than many other diseases, as there is no single diagnostic test or measurement, such as HbA1c for diabetes mellitus, or blood pressure for hypertension. Medical record review of all self-reported cases is time-consuming and expensive, so is not a practical case-finding method for most large epidemiological studies and is not feasible as a method of population monitoring. Most countries, including Australia, already routinely collect information on dispensed medications, hospital or emergency department presentations and service provision billing. A novel case-finding algorithm that can be applied on a large scale and with data analysed automatically using available sources, would improve incidence and prevalence estimates and facilitate large population studies of RA.

Some work has been done in various countries to formulate such a case-finding algorithm. Many of these attempts, however, have used impractical data sources for large-scale use, such as interviews with GPs or survey participants [10, 13], or have used administrative data (such as outpatient billing codes attached to a specific diagnosis or medical speciality) that are not available in Australia and many other contexts [14–20]. Others have not included medication dispensing [14, 16, 18], which is a known parameter that improves performance as discussed above, or were conducted prior to widespread use of biologics [21], or have not attempted to exclude other rheumatologic/autoimmune conditions [10, 15, 16, 19, 22, 23]. One used self-reported RA as the gold standard diagnosis, likely leading to many false positives [24]. One started from a very strict inclusion of use of bDMARDs plus a rheumatologist visit, which likely would have led to a high rate of false negatives [25], and another examined only hospital patients so was unable to capture a population start-point [26]. These studies are reviewed in detail in Additional File 1.

In this study we evaluated cases of self-reported RA in a large Australian population-based cohort, the Australian Longitudinal Study on Women’s Health (ALSWH). We developed case definitions that use self-reported diagnosis, self-reported medications, and administrative data on medications, hospital admissions and service provision. The aim of this study was to provide an algorithm that can be used for case-finding of RA to estimate RA incidence and prevalence that can be adjusted for the data sources available in many countries and to provide a more standardised way to conduct future research and data collection, monitor disease within populations, and interpret already collected data. We also aim to provide the first approximate cohort of RA in Australia that is not reliant solely on self-report.

## Patients and methods

### Participants

**Fig. 1:**
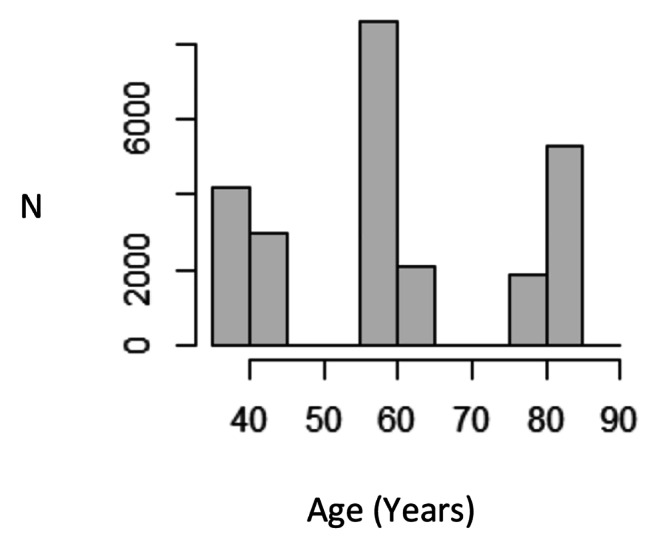
Histogram of the age of included participants at the time of the survey

The ALSWH is a prospective cohort study of 57 404 women living in all states and territories in Australia, initiated in 1996. Participants received surveys every three years from 1996, with the surveys ongoing to date. The ALSWH has four age cohorts. The original three cohorts were from birth years 1921-26, 1946-51 and 1973-78, and were randomly sampled from the Health Insurance Commission (Australia’s national health insurance system at that time) in 1995, with over-sampling of women from rural and remote areas. A fourth cohort of women from birth years 1989-95 was added in 2013 [27, 28]. The current study used survey data from survey 4 in 2005 of the cohort born in 1921-26 (then aged 79–84), surveys 5 (2007, 56–61 years) and 6 (2010, 59–64 years) of the cohort born in 1946-51 and survey 7 (2015, 37–42 years) of the cohort born in 1973-78. This constituted a total of 34 993 responses from 25 467 participants (Table 1; Fig. 1). Each participant was only included once. These surveys were selected for use because they asked participants questions about both self-reported diagnosis of RA and self-reported medications.

**Table 1:** Survey cohorts analysed for this study

Birth Year	Age at Survey	Surveys Used	Number of Respondents
1921-26	79–84	4	7 158
1946-51	56–61 (5) 59–64 (6)	5, 6	10 638 (5) 10 011 (6)
1973-78	37–42	7	7 186

### Data sources

The ALSWH survey data were linked to data from the Pharmaceutical Benefits Scheme (PBS), Australia’s national drug subsidy program. This program covers medications dispensed by community pharmacies and private hospitals, accounting for 75% of all prescriptions within Australia, and it is expected that it would cover more than 75% of prescriptions for RA medications due to their ongoing nature, the prescribing processes and cost. The PBS also covers discharge medications from public hospitals in all states and territories apart from New South Wales (NSW) and the Australian Capital Territory (ACT). The available PBS data covered the period May 2002 to June 2020. Survey data were also linked with International Classification of Disease (ICD) 10 codes for hospital and emergency department (ED) admission and discharge, which were available from 2007 onwards. We also looked at information from the Medicare Benefits Schedule (MBS), which is a list of health professional services that the Australian Government subsidises. MBS items provide patient benefits for a wide range of health services including consultations, diagnostic tests and operations. Linked MBS data were available for January 1996 to June 2020.

### Data linkage

Data were linked using a unique participant identification number, available for each woman in the ALSWH survey, which was attached to their PBS and ICD-10 data by the data management team of the ALSWH. Access to linked data was provided through a secure facility in Brisbane, Queensland, and in Newcastle, NSW.

### Data management

Participants with self-reported RA were identified in the ALSWH survey data by a positive response to the question “In the past 3 years have you been diagnosed or treated for RA?”. We divided medication case-definitions for RA into mid and strict (Table 2). The mid definition included patients taking prednisone/prednisolone or disease modifying anti-rheumatic drugs (DMARDs), including biologic, conventional synthetic and targeted synthetic. The strict definition excluded individuals taking only steroids. The search was conducted according to Anatomical Therapeutic Chemical Classification System (ATC) codes. Participants fulfilling each definition were identified based on self-reported medications, according to their response to the survey prompt “Please write down the names of all your medications, vitamins, supplements or herbal therapies”. The self-reported medication definition therefore included patients who self-reported medication use at the time of the included survey/s only.

**Table 2:** Anatomic & therapeutic classification (ATC) codes of medications included in medication definitions

ATC code	Drug name	Included in mid definition	Included in strict definition
L04AX03	methotrexate	√	√
A07EC01	sulfasalazine	√	√
L04AA13	leflunomide	√	√
M01CC01	penicillamine	√	√
L04AB01	etanercept	√	√
L04AB02	infliximab	√	√
L01XC02	rituximab	√	√
L04AB04	adalimumab	√	√
H02AB07	prednisone	√	×
H02AB06	prednisolone	√	×
M01CB01-05	gold preparations	√	√
L04AB05	certolizumab	√	√
L04AB06	golimumab	√	√
L04AA24	abatacept	√	√
L04AA29	tofacitinib	√	√
L04AA44	upadacitinib	√	√
L04AA37	baricitinib	√	√
L04AC07	tocilizumab	√	√
P01BA02	hydroxychloroquine	√	√

The linked data were then examined. PBS data of ATC codes were used to identify RA cases based on PBS-defined medication, according to the two definitions above (Table 2). The PBS definitions included patients taking the specified medications at any time point during the PBS data collection period. Limitations of the PBS database for this study are that prior to April 2012 payments below the co-payment threshold at which the PBS would cover part of the cost (up to $35.40) were not recorded, meaning that methotrexate, hydroxychloroquine, azathioprine and some other older conventional DMARDs were not captured, and medications dispensed solely to an inpatient in a public hospital are also not included, which we would expect to lead to some false negatives. ICD-10 codes for hospital and ED episodes were examined for each Australian state and territory. Both primary and secondary diagnoses coding were included. The ICD-10 codes M05 (rheumatoid arthritis with rheumatoid factor) and M06 (other rheumatoid arthritis) were selected as representing RA. ED coding was available from the ACT, NSW and Western Australia (WA) only, and participants were selected using the same ICD-10 codes as for hospital admission.

The medication definitions, self-reported RA, and admission/ED definitions were all examined as isolated methods for determining presence of RA. Combination of the different RA definitions, across the whole cohort series, was not possible due to varying years of data linkage.

To increase specificity by reducing the number of patients taking DMARDs for non-RA conditions, individuals with ATC codes for anti-psoriatic medications (D05) and intestinal anti-inflammatory agents (A07E) in either the PBS or self-reported medication data were excluded. Intestinal anti-inflammatory agents include locally acting corticosteroids (e.g. Rectal foams) and aminosalicylic acid and similar agents (e.g. Mesalazine). Anti-psoriatic agents include topical antipsoriatics (tars, antracen derivatives, psoralens and others), and systemic antipsoriatics (psoralens, retinoids and fumaric acid derivatives) (Table 3). Similarly, individuals with ICD-10 coded episodes corresponding to a number of other autoimmune or inflammatory conditions (Table 4), for which the DMARDs used in RA can also be used, were excluded for the same reason. Finally, those patients with MBS codes corresponding to services for inflammatory bowel disease (IBD) or psoriasis were excluded (Table 4). This exclusion was not applied to patients classified as RA based on admission/ED coding for RA as these were felt to have adequate specificity. This process was also applied to the self-reported RA group, using their linked administrative data, and the resulting group is referred to as the refined self-reported RA group.

**Table 3:** ATC codes used to exclude individuals using medications for psoriasis or inflammatory bowel disease

ATC Code	Description	Subcodes	Example Medications
D05	Antipsoriatics	D05A D05B	Tars Calcipotriol + betamethasone Psoralens for systemic use Retinoids for treatment of psoriasis
A07E	Intestinal anti-inflammatory agents		Corticosteroids acting locally Antiallergic agents Aminosalicylic acid and similar

**Table 4:** ICD-10 codes and MBS item numbers used to exclude patients with alternative diagnoses

Condition	ICD-10 _a_	MBS _b_
Ankylosing spondylitis	M45	
Myositis	M33	
Non-radiographic axial spondyloarthritis	M46·9	
Psoriatic arthritis	L40·5	
Enteropathic arthritis	M07·60	
Ulcerative colitis	K51·90	66,522, 66,523
Crohn’s disease	K50	66,522, 66,523, 63,740, 63,741, 63,743
Giant cell arteritis	M31·6	
Polyarteritis nodosa	M30·0	
Microscopic polyangiitis	M31·7	
Granulomatosis with polyangiitis	M31·3	
Eosinophilic granulomatosis with polyangiitis	M30·1	
Psoriasis		14,050

a: International Classification of Diseases, 10th Edition

b: Medicare Benefits Schedule

Those excluded from the original self-reported RA group during the refinement process and those remaining in the refined self-reported RA group were compared in terms of rurality, insurance status, and specialist access/review.

Prevalence of RA according to each definition was calculated using the appropriate denominator, i.e. the number of survey respondents was used for self-reported definitions, while the number of participants with a PBS record was used for PBS definitions, and the number of participants with any ED or hospital presentation for hospital/ED prevalence.

All data were managed in RStudio [29]. Missing values in the surveys (self-reported RA and self-reported medications) were handled by negative imputation.

## Results

**Fig. 2:**
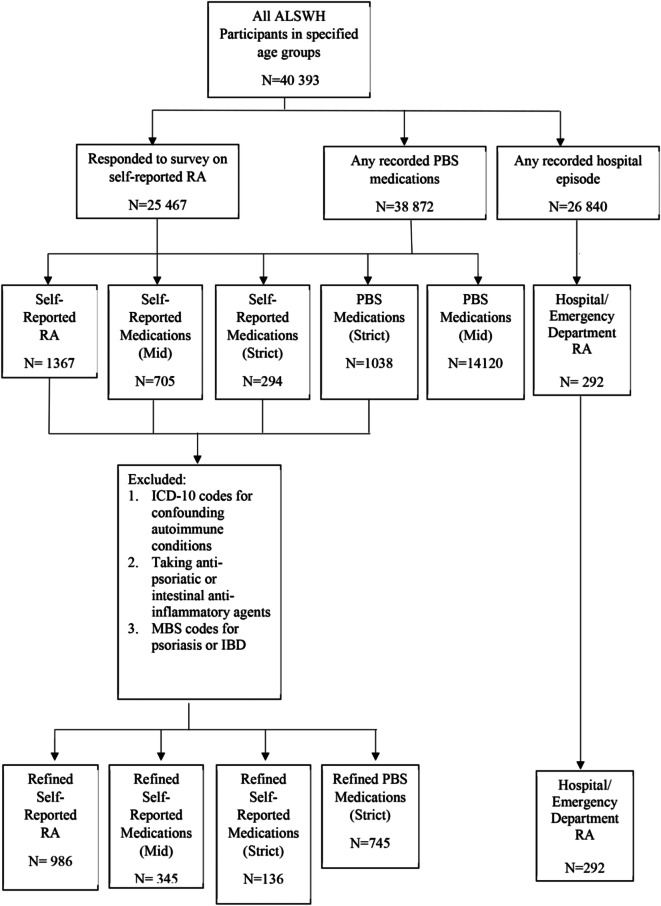
Process of forming and refining survey and administrative RA case definitions

**Fig. 3:**
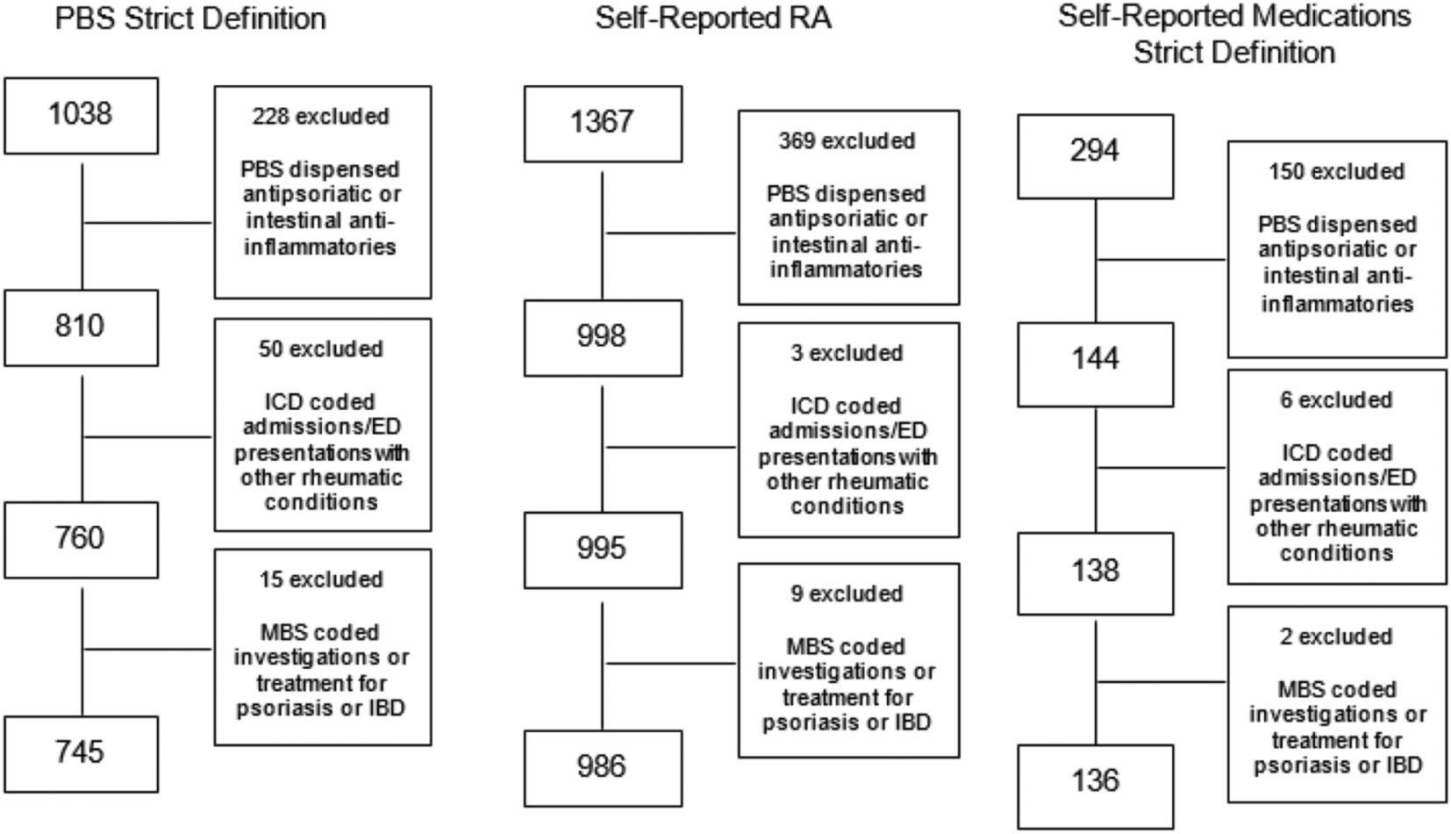
Case numbers excluded with each refinement step for the PBS strict medication definition, self-reported RA definition and self-reported medications strict definition

Of the 25 467 survey participants, 1367 (5.4%) women self-reported RA. 292 participants (1.1%) were coded as having RA based on hospital/ED records. 1038 women (2.8%) had PBS dispensed medications fulfilling the “strict” criterion. By self-reported medication definitions, there were 725 cases (3.3%) by the mid definition, and 294 (1.2%) by the strict definition. The PBS (mid) definition was excluded as an isolated method of defining RA due to high numbers of patients (*n* = 14,120) who had ever been prescribed prednisone/prednisolone, as expected given the breadth of uses for this medication. Following the refinement process described above (excluding those with coding for other potentially confounding conditions), case numbers decreased for all definitions. These numbers are shown in Fig. 2. The numbers of cases excluded at each step of the refinement process for the PBS strict definition, self-reported medication strict definition, and RA self-report are expanded in Fig. 3. Table 4 outlines the original and refined case numbers and prevalence.

**Table 5:** Original compared to refined RA case numbers according to definitions

Definition	Cases (Original) *n*	Prevalence % (95% CI)	Cases (Refined) *n*	Refined Prevalence % (95% CI)
Self-reported RA_a_	1367	5·4 (5·09 − 5·64)	986	3·9 (3·63 − 4·11)
Self-reported medications (strict)	294	1·2 (1·02 − 1·28)	136	0·5 (0·44 − 0·62)
Self-reported medications (mid)	725	3·3 (0·75 − 2·97)	345	1·4 (1·21 − 1·49)
PBS medications (strict)	1038	2·8 (2·51 − 2·83)	745	1·9 (1·78 − 2·06)
Admitted/ED_b_	292	1·1 (0·97 − 1·21)	N/A	N/A

a: Rheumatoid arthritis

b: Emergency department

The results of comparison between those excluded from the original RA group during refinement, and those remaining in the refined self-reported RA groups are outlined in Additional File 2. Those in the refined group were significantly more likely to report specialist review in the last 12 months compared to those in the excluded group *p* = 0.0004, no other differences were significant.

Finally, we checked correlation between cases defined by hospital/ED and dispensed medications. Of the 292 admitted/ED cases, only six individuals were not on any PBS recorded RA medications, of whom three also did not self-report RA.

### Role of the funding source

The study sponsors had no role in study design, collection, analysis and interpretation of data, in the writing of the report, nor in the decision to submit the paper for publication.

## Discussion

This Australian study supports the poor accuracy of self-reported RA as a sole measure of RA diagnosis, and strengthens the argument for finding a more accurate, yet practical, way to classify RA at a population level. The prevalence of unrefined self-reported RA in our study of 5.4% is much higher than would be expected for true RA, even among an all-female cohort aged over 34 years, in which the prevalence of RA is higher than in males or younger age groups [3, 30]. A study of 7443 post-menopausal women aged 50–79 years in the USA found a validated prevalence of 0.6% [9] and a study of French women aged 40–65 years found a validated prevalence of 1% [10], and we would expect our true prevalence to be similar.

We explored the use of available self-reported and administrative data to improve the accuracy of case-finding methods for RA. A question on medications is a relatively simple addition to population surveys and has been proposed to improve validity of self-reported disease. In the Black Women’s Health Study, the positive predictive value (PPV) of self-reported RA increased to 76% in women who reported taking DMARDs and to 61% in women who reported taking non-steroidal anti-inflammatory drugs (NSAIDs), compared to only 29% in women who did not report taking any related medications. When women using only prednisolone, or those reporting other rheumatic conditions, were excluded, and only those taking DMARDs were included, the PPV increased to 88% [11]. This suggests use of DMARDs as a case-finding method is likely to be relatively accurate. The current study developed two self-reported medication case definitions in keeping with this previous literature and found that excluding those taking only prednisone/prednisolone gave a prevalence of 1.2%, which is closer to the expected prevalence in our population [31, 32]. In contrast to the Black Women’s Health study, the effect of adding non disease specific medications like NSAIDs and steroids did not appear helpful in this study. This would be expected due to the breadth of indications for use of NSAIDs or steroids and therefore a lack of specificity for an RA diagnosis.

The PBS database provides a more complete and objective measure of medication prescribing than self-report. The PBS is one of the few medication reimbursement schemes in the world that provides whole population coverage. Additionally, in Australia nearly all medications for RA require prescription for access and are used according to PBS restrictions, meaning they are recorded on the PBS database. Use of PBS dispensed DMARDs as a sole method for case-finding (PBS-strict definition) gave a slightly higher than expected prevalence of 2.8%, which is not unexpected given the likely inclusion of some individuals taking DMARDs for other rheumatic or immune conditions, as the PBS did not record the indication for use. This is supported by the prevalence of 1.9% once individuals who had been admitted with, or had medications consistent with, other rheumatic/immune conditions were excluded. There was a discrepancy between self-reported and PBS strict medication definitions, with a much lower prevalence by the self-reported definition. This is likely influenced by a lower number of women answering this question, and under-recording of medications, such as methotrexate or injectable DMARDs that are not taken daily. The self-reported (strict) group was on average approximately 1 month older than the PBS dispensed (strict) group, apart from the young cohort where the mean age was approximately 1 month younger. We chose to apply the dispensed medication definitions to the total group rather than applying it in patients who additionally had self-reported RA, as we believe the established limitations of self-report as a diagnostic criterion would mean using this as a starting point for our case-definitions would go against the aims of this study. In addition, by using purely administrative data for the definitions and not requiring self-report/survey data, we have created a tool to approximate cases at a population level without use of intensive resources, making this of greater practical use.

Admission and ED data are likely to be the most specific measure of RA, and are of similar specificity to medical record review, which is usually held to be the gold standard. A recent study from Western Australia found that RA classified by ICD-10 discharge codes in hospital records had a sensitivity of 90% and PPV of 91% compared to rheumatologist medical record review [23]. In our study, the available data covered all public hospitals and EDs, and additionally covered private and day hospitals in some states, including NSW (the most populous Australian state). The accuracy of this definition is also supported by the strong correlation between admission/ED and PBS dispensed RA medication. The major limitation to using hospital data is poor sensitivity, as most people with RA do not require hospital treatment, and in Australia we do not have a population database that records diagnoses associated with public outpatient visits. This definition will therefore underestimate true RA prevalence. This likely contributes to a bias towards only more severe cases being included, or towards patients with more comorbidities, as they are more likely to have required admission or ED review. Reassuringly, however, in the current study, 292 cases were identified by this method, giving a prevalence of 1.1%, which is around the expected value for true RA. Given this, using the admitted or ED group is likely a good compromise for a well-validated RA cohort without performing individual medical record review.

MBS codes were used to improve specificity of medication-defined and self-reported cases. The MBS requirements were not applied to hospital-defined cases as these were felt to represent physician-diagnosed, and thus confirmed, cases that did not require further validation. The MBS provides specific item numbers for some diagnostic and service items, but is not comprehensive. Codes were available for diagnostic tests specific to IBD and treatment specific to psoriasis and these were used to exclude individuals with these conditions, which can cause non-RA inflammatory arthritis that can also require DMARD therapies. The MBS codes could not be used to identify individuals that had consulted a rheumatologist as service codes for consultations do not include clinician speciality.

The main limitation to our study is that we were unable to use medical record review, blood tests or physician review as the gold standard comparator due to restrictions imposed by the ethics approvals of the ALSWH and the survey data collection process. For this reason we were unable to statistically compare our case methods for accuracy relative to a gold or reference standard. However, access to the admission/ED data does provide a relatively well-validated group for comparison, as the diagnosis codes are provided based on clinician review. We also acknowledge that there is the potential for exclusion of some participants with true RA during the refinement process if these individuals had other concomitant inflammatory conditions. Our generalisability is limited to only women; however this cohort has been deliberately sampled to be representative of the total female Australian population (in the included age groups) so generalisability should be high within the female population. The true prevalence in the total population (male and female) would be expected to be lower than the numbers reported in our study, given the female preponderance of RA.

We propose two final case-definitions for use in further study of RA and its risk factors using the ALSWH data, a “Documented RA” group, using admitted/ED patients, and a “Treated RA” group, using ‘refined’ case definitions of PBS dispensed medications. The “Documented RA” group would be preferred when high specificity is essential, and the “Treated RA” group when sensitivity and broader generalisability is needed. Additionally, these definitions should be used to improve the national monitoring of RA in Australia, and with adjustments for local data sources, in many other nations. While we acknowledge that none of the methods to estimate RA cases is perfect, and the lack of comparison with a reference standard, the present study provides an algorithm for identifying RA cases that strikes a balance between improving accuracy and practicality/resource use. This provides a solution to the need for a more standardised and pragmatic method for RA definition to use in large studies and at a population level. If self-reported data are used, refining such a definition by excluding likely false-positive cases with the methods described above is likely to improve performance significantly.

## Conclusions

We provide a first approximate RA cohort in Australia that does not rely on self-report. We propose the use of two case-definitions for RA, a “Documented RA” group, using admitted/ED patients, and a “Treated RA” group, using refined PBS dispensed medications. These definitions could be used for future population studies and for ongoing monitoring of incidence and prevalence at the national level in Australia. With adjustment to the particular administrative data available in other countries, this algorithm could be applied to the broader global context. This study provides a practical solution to an unmet need in both Australia and internationally for a more standardised, yet resource-effective method to define RA on a large scale, using relatively easily obtainable input data that can be obtained at much lower cost than current gold standard methods.

### Electronic supplementary material

Below is the link to the electronic supplementary material.

Supplementary Material 1

Supplementary Material 2

Supplementary Material 3

## Data Availability

Due to ethics approvals and confidentiality the datasets supporting the conclusions of this article are not publicly available. Will individual participant data be available (including data dictionaries)? Not available. What data in particular will be shared? Not available. What other documents will be available? Analytic code. When will data be available (start and end dates)? Immediately following publication, ending 3 years after publication. With whom? Researchers who provide a methodologically sound proposal. For what types of analyses? To achieve aims in the approved proposal. By what mechanism will data be made available? Proposals should be directed to the corresponding author

## Abbreviations

RA: Rheumatoid arthritis
ALSWH: Australian Longitudinal Study on Women’s Health
ED: Emergency department
ICD: International Classification of Diseases
PPV: Positive predictive value
GP: General practitioner
DMARDs: Disease modifying antirheumatic drugs
bDMARDs: Biologic disease modifying antirheumatic drugs
PBS: Pharmaceutical Benefits Scheme
NSW: New South Wales
ACT: Australian Capital Territory
MBS: Medicare Benefits Schedule
ATC: Anatomical Therapeutic Chemical Classification System
WA: Western Australia
IBD: Inflammatory bowel disease
SA: South Australia
HREC: Human Research Ethics Committee
USA: United States of America
NSAIDs: Non steroid anti-inflammatory drugs
