# Supplementary material for: A novel method to monitor rheumatoid arthritis prevalence using hospital and medication databases
Source: Arthritis Res Ther. 2024 Jul 16;26:133. doi: 10.1186/s13075-024-03366-x (PMC11251372; doi:10.1186/s13075-024-03366-x)
Supplement: Supplementary file 1 — Supplementary Material 1 [file 13075_2024_3366_MOESM1_ESM.docx]

Additional File 1. Appraisal of previously conducted validation studies

| **Author, Year** | **Population** | **Sample Size** | **Data Sources** | **Gold Std/Validation** | **Case Definitions Used** | **Strengths** | **Weaknesses** | **Prevalence** |
| --- | --- | --- | --- | --- | --- | --- | --- | --- |
| *Carrara 2014*  Case-control & cohort diagnostic accuracy study | Training set: Random sample of 900 outpatients >16 years from single tertiary rheumatology clinic in Pavia, Lombardy, Italy  Validation set: random sample from single rheumatology outpatient clinic,  all participants from six primary care physicians of Pavia, Lombardy, Italy | Training set: 900 (300 cases, 600 controls)  Validation set: 6225 | Administrative health record of all resident population  Archive of chronic disease certification for co-payment exemption  Hospital discharges of public/private hospitals  All outpatient drug prescriptions reimbursable under National Health Service (NHS) | Clinical diagnosis from medical record reviewed by single independent investigator | RA certification by rheumatologist  Absence of certification for other autoimmune disease (AID)  Code for rheumatoid arthritis (RA) in hospital discharge  Any disease modifying anti-rheumatic drug (DMARD)  (individually & in combination) | Able to validate using medical record review with good sensitivity (92.5%) and specificity (99.8%)  Excluded other AID | Does not cover prescriptions for residential aged care facility (RACF) pts  Requires availability of coding by rheumatologist  Only in single region in Italy (diversity/representativeness) | 0.3% male and 0.73% female |
| *Kubota 2021* | Random sample of 19374 pts who received hospital care in 64 hospitals of Tokushukai Medical group Japan  With follow up >365 days | 19734 pts had algorithm applied  333 possible RA patients had medical record reviewed  Final result N=73 | Elec database routinely collected by hospital: includes condition codes, drugs used in inpt/outpt care, procedure codes | Medical record review of possible RA cases by 2 rheumatologists | RA condition code + any DMARD  OR  Oral steroid + no other systemic AID | High sensitivity (~73%) & positive predictive value (PPV) (80%)  Hospitals from across Japan - more representative  Excluded other AIDs + looked at medications | Only patients treated in hospital (although does include hospital outpatients)  Able to validate using medical record  Unable to capture from population start-point | 0.56% |
| *Ter Wee 2021* | Random sample of 55-85 year olds (in 1992) from 11 municipalities in Netherlands with surveys every 3 years  Used survey 2005-2006 | 1908 pts  With subsample of 300 for validation | Patient survey  Questionnaire to general practitioners (GPs) including question re prescribed drugs | Medical record review of GP record | Self-reported RA + either:  Self-reported specialist review (sensitivity 60%, specificity 99%)  GP-reported DMARD use (sensitivity 100%, specificity 97%)  or both (sensitivity 60%, specificity 100%) | Validation through examination of medical record  Geographic diversity within Netherlands  Could be adjusted to use only self-reported variables which would be practically easy | Limited all diagnoses to self-reported RA  Used self-reported medications only  Requires GP visits and separate questionnaire so limited applicability on large scale  Did not use administrative data  Did not exclude other AIDs  Elderly patients only  Using GP records not specialist review, may not be up to date/may be inaccurate | N/A |
| *Hanly 2015* | Nova Scotia, Canada residents enrolled in Medical Services Insurance April 1997-March 2011 (excludes armed forces and Native Canadians), who had also been seen at Arthritis Centre of Nova Scotia | 535 cases matched with 2140 controls | Administrative database covering physician speciality, diagnostic code, hospital discharge code, diagnostic codes  Medical record from Arthritis Centre | Rheumatologist diagnosis in record of Arthritis Centre | 7 options:  2 physician coded visits RA 2 months apart (sensitivity 83%, specificity 815)  As above but excluding other AIDs (sensitivity 69%, specificity 81%)  3x RA diagnostic billing codes (sensitivity 83%, specificity 82%)  1x RA hospitalisation (sensitivity 21%, specificity 99%)  1x rheumatologist coded RA (sensitivity 88%, specificity 76%)  Combination (Sensitivity 92%, specificity 74%) | Excluded other AIDs  Had excellent data sources which are easy to access in this context | Single centre study from Capital Health District  Did not use medications  Validated on rheumatology clinic population (ie not on general population)  Access to this level of data not available in many countries | 10.6% (in a rheumatology clinic cohort) |
| *Thomas 2008* | General Practice Research database (GPRD), covers 5% of UK population, chosen practices are broadly representative of all practices | 400 | GPRD- outcomes of consultations, hospitalisation, specialist referrals, test results, prescriptions | Rheumatologist review of GP medical record | Final model:  >1 RA code (sensitivity 80%, specificity 81%), 1+ DMARD prescription with no prior alternative indication (sensitivity 78%, specificity 96%), No alternative AID diagnosis (sensitivity 86%, specificity 40%) | Good overview of general population, used administrative data rather than questionnaires or self-report | Pre-biologic era  Only included practices and patients who agreed may have biased results  Required access to GP record | N/A |
| *Widdifield 2013* | Random sample of patients >20 years old seen at least twice between March 2009-2011 from 18 rheumatology clinics in Ontario, Canada | 450 | Administrative data from April 1991 to April 2011;  Physician claims + diagnosis + physician speciality on universal health insurance claims database, hospital and outpatient discharges with diagnosis codes  Medication data (pharmacy claims) available only for >65 years old | Rheumatologist recorded diagnosis in medical record - extracted by single blinded reviewer | Tested >130 case finding algorithms using combinations of data sources.  Optimal algorithm:  1 hospitalisation code ever OR 3 physician diagnosis codes + 1+ by specialist in last 2 years (sensitivity 97%, specificity 85%) | Tested for exclusion of other AIDs  Access to medical record review  Universal coding/insurance data available | Noted high number of false positives when using specialist diagnosis codes, likely due to high number of rheumatologists coding for disease even if diagnosis uncertain  Requires access to physician billing diagnoses + speciality  Medications only tested in small number >65 years old | 33% (in rheumatology clinic population) |
| *Almutairi 2021* | Random selection of medical records of patients with International Classification of Diseases (ICD-10)coding M05 and M06 at largest tertiary hospital in Perth, Western Australia | 200 patients sampled, 87 had record review completed (remainder excluded due to missing data) | Hospital discharge codes, medication charts and paper based notes | Medical record review by 2 independent clinicians, disagreement decided by 3rd independent reviewer– documented diagnosis of RA + EULAR 2010 criteria | Excluded patients with 2 subsequent health contacts for other arthritis, systemic lupus erythematosus (SLE) or connective tissue disease (CTD)  For discharge code alone PPV 92%, using 2+ primary RA diagnosis codes including bDMARD infusion codes PPV increased to 97.9%  bDMARD infusion codes had higher specificity but less sensitivity than primary diagnosis codes | Used easily accessible data only  Good gold standard  Excluded other AIDs | Only had access to inpatient diagnoses and medications so not applicable on population level  Relatively small sample size | N/A |
| *Kim 2011* | Medicare beneficiaries aged >65 years old with pharmacy benefits in Pennsylvania, USA (qualify for program by being a low-moderate income earner) with records from 2004-2008 | 9482 invited to participate, final population for record review n=157 | Medicare claim codes for RA, also lists which specialists have seen, inpatient/outpatient/procedure (radiology, laboratory) | Independent medical record review by ‘several’ rheumatologists, either documented diagnosis of RA by rheumatologist or fulfilment of 1987 diagnostic criteria | 2+ claims for RA (PPV 55.7%), 3+ claims for RA (PPV 65.5%),  Adding 1+ DMARD prescription increased PPV to 86.2% for the 2+ claims algorithm,  Optimal algorithm: 2+ RA claims by rheumatologist + 1+ DMARD prescription (PPV 88.9%) | Used medications in combination with diagnosis codes  Good gold standard  Optimal algorithm had fairly good PPV | Needed to have claims data for specialist + diagnosis  Restricted population geographically and demographically  Didn’t exclude other AIDs | Not reported |
| *Cho 2013* | Korean national health insurance claims database (covers 100% of population), all patients >16 years old with diagnosis of seropositive RA between July and December 2009, excluding Medicaid patients & those from hospitals not enrolled in Copayment program | 59 823 | Demographic data, physicians, hospitals, comorbidities (diagnostic codes), prescribed medications | Enrolment in individual copayment beneficiaries program - requires official diagnostic report from referring doctor with seropositive RA fulfilling 1987 ACR criteria | Final algorithm: RA code + 1+ prescription claim for any DMARD within a year, (sensitivity 94.5%, PPV 92.4%, accuracy 90.3%) | Tested multiple medication combinations  Final algorithm performed well against gold standard  Large sample size  Diverse population within Korea  Data sources should be reasonably easy to adapt to other contexts | Seropositive RA only (more homogenous population)  Did not try to exclude other AID diagnoses  Final algorithm would not include patients not on treatment during study period |  |
| *Convertino 2021* | Tuscan universal public health system database from 2004 to April 2019,  Extracted those with first ever biologic DMARD (bDMARD) dispensing 2014-2016 and who had 1+ visit to Rheumatology Unit of Pisa University Hospital from 2013 to April 2019 | 277 | Inpatient and outpatient medication supply, exemptions from copayments database, hospital discharge records, ED access records, outpatient records for specialist visits | Medical record review documenting RA diagnosis | Four algorithms:  1- ICD-9 coded RA on hospital discharge or ED (sensitivity 0.53, specificity 0.89, PPV 0.74)  2- RA coded on copayment exemption (sensitivity 0.77, specificity 0.90, PPV 0.82)  3- Definition 1 AND 2 (sensitivity 0.37, specificity 0.95, PPV 0.81)  4- Definition 1 OR 2 (sensitivity 0.93, specificity 0.84, PPV 0.78) | Combination of medications and discharge information likely to be relatively widely available and applicable to many contexts | Started from very limited population (ie bDMARD + rheumatology clinic visit)  Single region in Italy only  Did not attempt to exclude other AIDs  Relatively small sample size | Not reported |
| *Waldenlind 2014* | Random extraction of patients from National Patient Register with one listed ICD10 code for RA between 2005-2008 at non primary outpatient care facility at Karolinska University Hospital in Stockholm Sweden (covers ~13% of population of Sweden) | 211 | National Patient Register of hospital discharges (100% coverage), non primary outpatient care (87% coverage)  Prescribed drug register (close to 100% coverage) of all drugs dispensed on outpatient prescription | Medical record review of 2010 EULAR /1987 ACR criteria | Incident group:  Base- First ever rheumatologist visit in 2008 with RA diagnosis code (86% correctly coded)  Strict - 2nd hospital visit for RA within 1 year AND no DMARD treatment for >6 months prior to first RA visit (91% correctly coded)  Prevalent group:  2+ visits to rheumatologist between 2005 and 2008 coded for RA (91% correctly coded) | Unique to study incident vs prevalent RA case definitions  Excellent population coverage of data sources  Very solid gold standard definition | Only used medication to exclude pre-existing diagnosis not for RA definition  Did not exclude other AIDs  Required rheumatologist coding of RA which is not widely available  Started from base population of tertiary hospital patients with RA coding | N/A  (NB didn’t report sensitivity, specificity, PPV) |
| *Widdifield 2014*  *Note: as per other Widdifield study but validation in primary care population rather than rheumatology clinic population* | Random sample of patients from 83 rural and urban physicians in Ontario, Canada aged 20+ years on 31/12/2010, who had valid health insurance, 1+ physician visit in previous year | 9500 | EMRALD primary care clinic database + administrative data from April 1991 to April 2011 (physician billing codes and diagnosis, hospitalization and ED encounters, hospital based outpatient clinics  Pharmacy claims for patients >65 years old | Medical record review for RA diagnosis / specialist review and medications by a single trained abstractor, with 10% checked for agreement by second abstractor | Best performing algorithm:  1 hospitalisation RA code OR 3 physician RA diagnosis code claims with 1+ by specialist in 2 year period (sensitivity 78%, specificity 100%, PPV 78%)  Ie same algorithm as in other Widdifield study | Very large sample size  Excluded other arthritis and AIDs  Tested >100 algorithms with combinations of data | Medications only available for >65 year olds  Requires physician diagnoses/billing codes which are not available in all contexts | 0.9%-1.3% (depending on strictness of reference standard case definition) |
| *Nguyen 2019* | E3N cohort prospective cohort study of women living in France and covered by a national insurance scheme primarily used by teachers aged 40-65 at inclusion, ongoing since 1990  Follow up per questionnaire 83% and loss to follow up <3% at 2014 | 3230, with 2182 returning questionnaire | Self-reported RA at 3 survey time points  Drug reimbursement claims since 2004  Follow up specific questionnaire in 2017 | Medical record review of subset of 399 patients who returned questionnaire by 2 independent blinded reviewers based on medical opinion rather than diagnostic criteria fulfillment | Self-report + immune related disease (IRD) questionnaire (sensitivity 94%, specificity 83%, PPV 72%)  Self-report + 1+ reimbursement RA medication (sensitivity 71%, specificity 87%, PPV 90%) | Large sample size  Use of self-report + medications is a feasible case definition for large scale study and had acceptable performance  Sampled from a large population cohort from across France | Used pts with self-reported ankylosing spondylitis as control group  Started from self-reported RA group  Use of questionnaire is not practical on large scale/population level  Didn’t exclude other AIDs | 0.97% |
| *Callhoff 2022* | Random sample from 6.6million insurants aged 18-79 in 2013 of a large German statutory health insurance company with outpatient ICD-10 code for RA in at least two quarters of 2013 | 6193, of which 3184 responded to questionnaire and 3140 provided consent for linking | Outpatient claims data with diagnosis code  Self-reported questionnaire (2015)  Medication claims of DMARDs, inflammatory markers and rheumatologist visit claims data | Self-reported RA | 1) 2x ICD codes for RA within year (PPV 81%)  2) 1) AND inflammatory markers (PPV 82%, sensitivity 84%, specificity 24%)  3) 1) AND medication (PPV 89%, sensitivity 57%, specificity 69%)  4) 1) AND rheumatologist visit (PPV 85%, sensitivity 55%, specificity 59%) | Looked at longitudinal consistency of diagnosis  Using medication + ICD-10 codes was best algorithm which is largely accessible data in many contexts | Used self-reported RA as gold-standard  Higher response rate to survey in those coded for RA (ie selection bias) | N/A |
| *Booth 2021* | 2004, 2008 and 2012 waves of HRS- a nationally representative longitudinal study of US residents aged over 50 years  Participants were 65 years or older in 2004, with linked Medicare data, had Part A and B Medicare coverage and responded to question about self-reported RA, excluding prevalence RA in 2004 | 3768 | Self-reported RA & medications  Billing claims from Medicare (inpatient, outpatient visits) with linked diagnoses using 4 year lookback period | Self-reported RA | 1- 1+ RA claims in primary/secondary diagnosis (sensitivity 0.23, specificity 0.92, PPV 0.16)  2- 2+ RA claims within 2 year period (sensitivity 0.40, specificity 0.92, PPV 0.12)  3- 2+ RA claims, one from rheumatologist (sensitivity 0.55, specificity 0.91, PPV 0.05) | Looked at self-report vs self-report + self-reported medications  Used data easily available within USA, working around limitations with data availability | Needs data on rheumatologist visits  Didn’t exclude other AIDs  Used self-reported rather than dispensed/prescribed medication data  Not a nationally representative sample due to requirement for Medicare | 9.2% based on self-report, 6.2% based on single RA claim |
