# Supplementary material for: A novel method to monitor rheumatoid arthritis prevalence using hospital and medication databases
Source: Arthritis Res Ther. 2024 Jul 16;26:133. doi: 10.1186/s13075-024-03366-x (PMC11251372; doi:10.1186/s13075-024-03366-x)
Supplement: Supplementary file 2 — Supplementary Material 2 [file 13075_2024_3366_MOESM2_ESM.docx]

Additional File 2. Comparison of selected characteristics of excluded vs remaining self-reported RA individuals following refinement process

_
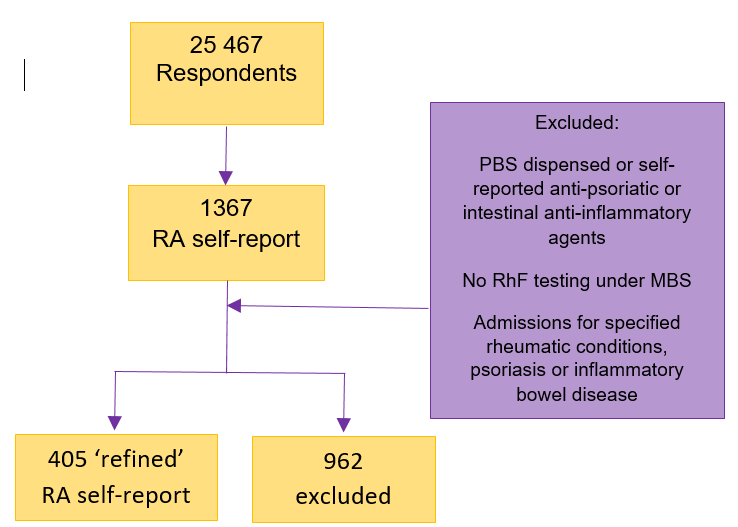
_

| **Variable** | **Refined**  N (%) | **Excluded**  N (%) | **P Value** |
| --- | --- | --- | --- |
| Private Insurance/DVA^a^ | 262 (64.7) | 584 (60.7) | 0.1855 |
| Healthcare Card | 204 (50.4) | 517 (53.7) | 0.2798 |
| Specialist access- poor | 70 (17.3) | 190 (19.8) | 0.3243 |
| No specialist consult last 12 months | 145 (36.0) | 446 (46.4) | 0.0004 |
| Living in major/inner centre (ARIA^b^ classification) | 322 (79.5) | 739 (76.8) | 0.309 |
| Living in outer/remote/very remote area (ARIA^b^ classification) | 90 (22.2) | 240 (25.0) | 0.3144 |
| Lives urban (RRMA^c^ classification) | 174 (43.0) | 378 (39.3) | 0.2292 |
| Lives rural/remote (RRMA^c^ classification) | 237 (58.5) | 600 (62.4) | 0.2027 |
| Lives urban or large rural (RRMA^c^ classification) | 225 (55.6) | 500 (52.0) | 0.2494 |
| Lives small rural/remote (RRMA^c^ classification) | 187 (46.2) | 488 (50.7) | 0.1932 |
| ^a^ DVA: Department of Veteran’s Affairs  ^b^ ARIA: Accessibility/remoteness index of Australia  ^c^ RRMA: Rural, remote and metropolitan area  All analyses done using Pearson’s Chi Squared Test with Yates’ continuity correction | | | |
