# Supplementary material for: A novel method to monitor rheumatoid arthritis prevalence using hospital and medication databases
Source: Arthritis Res Ther. 2024 Jul 16;26:133. doi: 10.1186/s13075-024-03366-x (PMC11251372; doi:10.1186/s13075-024-03366-x)
Supplement: Supplementary file 3 — Supplementary Material 3 [file 13075_2024_3366_MOESM3_ESM.docx]

**Additional File 1.**

**File name:** AdditionalFile1.docx

**Title:** Appraisal of previously conducted validation studies

**Description:** Table of author/year, population, sample size, data sources, gold standard/validation method, case definitions used, strengths, weaknesses and prevalence of previously published validation studies.

**Additional File 2.**

**File name:** AdditionalFile2.docx

**Title:** Comparison of selected characteristics of excluded vs remaining self-reported RA individuals following refinement process

**Description:** Flow chart showing refinement process and numbers for self-reported rheumatoid arthritis (RA) group. Table showing number, percentage and p values comparing the respondents remaining after refinement with those excluded during refinement in terms of selected sociodemographic variables. These data are also presented in graphical form.
